# *Arabidopsis thaliana* Genes Associated with *Cucumber mosaic virus* Virulence and Their Link to Virus Seed Transmission

**DOI:** 10.3390/microorganisms9040692

**Published:** 2021-03-27

**Authors:** Nuria Montes, Alberto Cobos, Miriam Gil-Valle, Elena Caro, Israel Pagán

**Affiliations:** 1 Unidad de Fisiología Vegetal, Departamento Ciencias Farmacéuticas y de la Salud, Facultad de Farmacia, Universidad San Pablo-CEU Universities, Boadilla del Monte, 28003 Madrid, Spain; nuria.montes.casado@gmail.com; 2 Servicio de Reumatología, Hospital Universitario de la Princesa, Instituto de Investigación Sanitaria (IIS-IP), 28006 Madrid, Spain; 3 Centro de Biotecnología y Genómica de Plantas UPM-INIA and Departamento de Biotecnología-Biología Vegetal, E.T.S. Ingeniería Agronómica, Alimentaria y de Biosistemas, Universidad Politécnica de Madrid, 28045 Madrid, Spain; alberto.cobos@upm.es (A.C.); miriam.gilvalle@gmail.com (M.G.-V.); elena.caro@upm.es (E.C.)

**Keywords:** *Arabidopsis thaliana*, *Cucumber mosaic virus*, genome-wide association studies, plant–virus interaction, seed transmission, virulence

## Abstract

Virulence, the effect of pathogen infection on progeny production, is a major determinant of host and pathogen fitness as it affects host fecundity and pathogen transmission. In plant–virus interactions, ample evidence indicates that virulence is genetically controlled by both partners. However, the host genetic determinants are poorly understood. Through a genome-wide association study (GWAS) of 154 *Arabidopsis thaliana* genotypes infected by *Cucumber mosaic virus* (CMV), we identified eight host genes associated with virulence, most of them involved in response to biotic stresses and in cell wall biogenesis in plant reproductive structures. Given that virulence is a main determinant of the efficiency of plant virus seed transmission, we explored the link between this trait and the genetic regulation of virulence. Our results suggest that the same functions that control virulence are also important for CMV transmission through seeds. In sum, this work provides evidence of a novel role for some previously known plant defense genes and for the cell wall metabolism in plant virus interactions.

## 1. Introduction

Viruses are major plant pathogens due to the detrimental effect of their infections on the host (i.e., to their virulence) [1]. Indeed, they have great impact on agronomic production worldwide, being the second most important cause of economic losses in crops only behind fungi [2] and accounting for the largest fraction of plant emerging diseases [3]. Hence, understanding the genetic basis of virulence in plant–virus interactions is central in minimizing the damage of virus epidemics.

Although virulence can be intuitively viewed as a pathogen-controlled trait, a large body of evidence indicates that in plant–virus interactions it is modulated by both host and pathogen genetic determinants [4,5,6,7]. Plant virus genes and even single mutations determining virulence have been extensively characterized, e.g., [8,9]. In parallel, plant genetic determinants have been also studied although at a lesser extent [8,10,11]. Interestingly, most of the works on the “host side” addressed the question from a plant pathology perspective. In this context, virulence is often defined either as the virus capacity to gain entrance to the plant or as the virus ability to induce symptoms, generally in the vegetative structures [12]. On the one hand, the use of the former definition of virulence led mostly to the identification of plant genes conferring resistance/immunity to virus infection, such as those related to the plant hypersensitive response, RNA silencing machinery or systemic acquired resistance [13,14,15]. On the other hand, host genes regulating virus cell-to-cell and long-distance movement, and plant photosynthesis and development [5,11,16] have been identified as virulence determinants when symptom severity was used as a proxy. Notably, most of these studies quantified virulence as a qualitative trait (susceptibility *vs*. immunity and mild *vs*. severe symptoms), which may limit the power of the approach to identify genetic determinants by reducing the information contained in qualitative variables as compared with analyses based on quantitative ones [17]. Moreover, virulence is also manifested in other plant traits, such as their ability to reproduce and survive, which are often not considered in the above-mentioned studies [18] but are relevant in agricultural settings and central in other ecological contexts in which virus infections are also commonplace.

Accumulating data indicates that, besides being of agronomic importance, plant viruses are also major ecological agents in wild ecosystems. Viral infections can drastically reduce the number of individuals in wild plant populations by decreasing the competitive or reproductive abilities of infected individuals [3,19,20]. As a consequence, wild plants evolved quantitative resistance and tolerance in response to infection [21,22,23], which suggests that viruses shape the genetic composition of the host population. Indeed, there is evidence that virus infections may act as a selective force for wild plant populations [23]. In wild ecosystems, the main impact of virus infections on the host population is through their detrimental effect on plant fitness [1]. Thus, analyses of virulence from an ecological or evolutionary perspective generally used the effect of infection on plant progeny as a proxy, but very little is known on the host genes regulating the effect of virus infection on plant fitness [18,24]. Certainly, the development of plant symptoms in vegetative structures may potentially impact plant fitness and it is likely that both traits have genetic associations [10]. However, the effect of infection on plant growth and on plant reproduction are not necessarily linked, e.g., [20,25,26]. Hence, the host genetic determinants of virulence as the effect of virus infection on plant progeny production are still poorly understood.

Under this definition, virulence has a direct impact not only on the host fitness but also on the pathogen’s. For instance, for plant viruses that are horizontally transmitted through insect vectors or by contact, it affects the number of available hosts [1]. More importantly, seed production is a major fitness component for vertically transmitted plant viruses, which accounts for at least 25% of all known species [27]. Indeed, we have recently shown that virulence is one of the main determinants of *Cucumber mosaic virus* (CMV) and *Turnip mosaic virus* (TuMV) seed transmission rate in *Arabidopsis thaliana* [28]. These results suggest that these two traits might have at least partially overlapping genetic regulations, although such link has not been explored yet. 

To address these questions, we utilized the interaction between *Cucumber mosaic virus* (CMV, *Bromoviridae*) and *Arabidopsis thaliana* (from here on “Arabidopsis”, Brassicaceae) as model. CMV is the plant RNA virus with the broadest host range, infecting about 1200 species in more than 100 plant families, including Arabidopsis [29]. This virus is horizontally transmitted by aphids and in Arabidopsis is also seed transmitted with an efficiency that depends on the host per virus genotype x genotype interaction, indicating that the host is involved in controlling this trait [28]. CMV is commonly found in wild populations of Arabidopsis at up to 80% prevalence [21], and therefore the Arabidopsis–CMV interaction is relevant in nature. Indeed, recent work strongly suggests that CMV infection selects for defenses in Arabidopsis populations of the Iberian Peninsula [23]. This geographic region has been shown to contain the largest Arabidopsis genetic diversity in Eurasia due to its role as refugia during the last glaciations [30,31]. Accordingly, substantial genetic variation has been described for relevant adaptive traits including diversity for responses to pathogens [23,32,33,34]. For instance, the infection of ten Iberian Arabidopsis genotypes, representing the variation of the species in this region, with different CMV and TuMV strains showed that virulence (quantitatively measured as the effect of infection on viable seed production) is also controlled by both virus and host genetic determinants [4,23,26].

In this work, we aim at characterizing the Arabidopsis genetic determinants associated with the effect of CMV infection on plant progeny production as a measure of virulence, and to explore the link between the generic control of virulence and of CMV seed transmission rate. To do so, we performed a genome-wide association study (GWAS) with 154 Arabidopsis genotypes from the Iberian Peninsula, for which annotated genomes are publicly available [35] and using Arabidopsis seed production in infected and non-infected plants as the quantitative relevant trait. We further investigated whether the identified genetic determinants of virulence play also a role in modulating CMV seed transmission rate.

## 2. Materials and Methods

### 2.1. Plant Material

**Figure 1:**
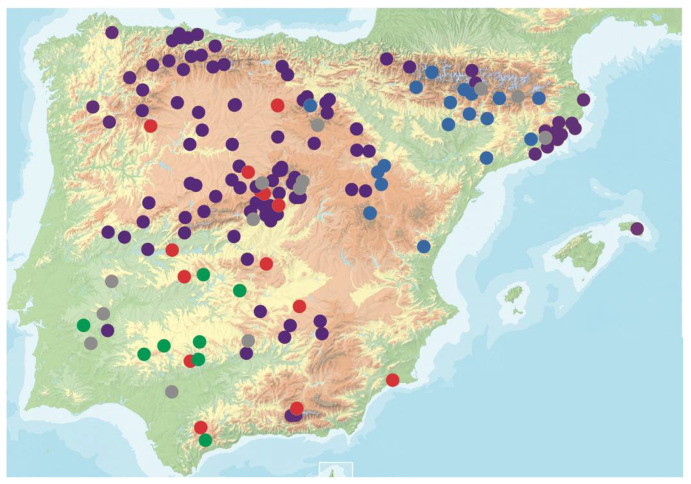
Geographic distribution of *Arabidopsis thaliana* genotypes analyzed in this study. Circles indicate population locations. Colors indicate genetic groups as defined in [39] (group 1: purple, group 2: blue; group 3: red; group 4: green). Grey dots indicate genotypes eliminated from the analysis.

We used 165 Arabidopsis genotypes from the Iberian Peninsula (Figure 1 and Table S1). Genotypes were collected from different populations and selected to cover the genetic and environmental diversity of the species in the region [36,37]. This collection spanned 800 km × 700 km, populations being spaced 384.9 ± 3.7 km on the average (from 20.2 to 1038.1 km). Altitudes ranged from 123 to 1670 m above sea level. Each sample was genetically different based on previous single nucleotide polymorphism (SNP) genotyping and genome sequences [38,39]. Similar sets of Arabidopsis genotypes have been previously shown to be powerful for fine mapping of genomic regions associated with natural variation of quantitative traits, such as plant life history traits, including seed weight [40], seed dormancy [41], and flowering time [39] both under field and greenhouse conditions. 

All genotypes used in this study were propagated by selfing during two generations by the single seed descent procedure, in a glasshouse supplemented with lamps to pro- vide a long-day photoperiod. This allowed for the reduction of residual heterozygosity that might contain some wild individuals but also removed any potential maternal and grand-mother effects. Seeds were stratified (darkness, 4 °C) for 7 days before germination at 25/20 °C day/night, 16 h light in a greenhouse. Ten-day-old seedlings were transferred to 4 °C, 8 h light, for vernalization for 8 weeks. After vernalization, plants were transplanted to 0.43 L pots containing a 3:1 peat-vermiculite mix and returned to the greenhouse, where they were kept at 25/20 °C day/night and 16 h light (intensity, 120 to 150 mol s/m^2^) until the end of the experiment. 

### 2.2. Virus Isolate and Inoculation

Fny-CMV (GenBank accession numbers NC_002034, NC_002035 and NC_001440) was derived from biologically active clones [42] by in vitro transcription with T7 RNA polymerase (New England Biolabs, Ipswich, MA, USA), and transcripts were used to infect *Nicotiana benthamiana* plants for virus multiplication. Six Arabidopsis plants per genotype were mechanically inoculated with *N. benthamiana* CMV-infected tissue ground in a solution containing 0.1 M Na_2_HPO_4_, 0.5 M NaH_2_PO_4_, and 0.02% DIECA (0.01 M phosphate buffer (pH 7.0), 0.2% sodium diethyldithiocarbamate), and four plants per genotype were mock-inoculated with inoculation buffer. Inoculations were carried out when plants were at developmental stages 1.05 to 1.06 [43]. After inoculation, all individuals were randomized in the greenhouse. The efficiency of inoculations was determined by detecting virus presence in all plants. To do so, 15 days post-inoculation three disks with a diameter of 4 mm were collected from different systemically infected rosette leaves. From these plant samples, total RNA extracts were obtained using TRIzol^®^ reagent (Life Technologies, Carlsbad, CA, USA), and 10 ng of total RNA was added to a Brilliant III Ultra-Fast SYBR green qRT-PCR master mix (Agilent Technologies, Santa Clara, CA, USA) according to the manufacturer’s recommendations. Specific primers were used to amplify a 154-nt fragment of the CMV MP gene [28]. Each plant sample was assayed in duplicate on a LightCycler 480 II real-time PCR system (Roche, Indianapolis, IN, USA). The rate of inoculation success was 98.8%. A similar procedure was used to confirm CMV absence in mock-inoculated plants.

### 2.3. Quantification of Virulence 

Seeds were harvested at complete plant senescence, and the total seed weight per plant (*SW*) was obtained for infected and mock-inoculated plants. Virulence (*V*) was estimated as 1 minus the ratio of the total seed weight of each infected plant (*SW_i_*) to the averaged total seed weight of mock inoculated (*SW_m_*) plants from the same genotype.

Seed viability was measured as the germination percentage of 200 seeds per plant. Germination assays were carried out at least 60 days after harvesting to avoid differences on seed dormancy. Relative differences in seed viability between infected and control plants were used to correct *V* values such that only viable seed production was reflected.

### 2.4. Efficiency of Virus Seed Transmission

The efficiency of CMV seed transmission was estimated as the percentage of infected seeds that gave rise to infected progeny in grow-out tests. One-hundred seeds per replicate were washed in a 10% bleach solution. Then, seeds were placed into Petri dishes containing Murashige–Skoog medium, stratified for 5 days at 4 °C, and germinated in a growth chamber at 22 °C, under 16 h of light (intensity, 120 to 150 mol s/m^2^). Following [28], seedlings at 15 days poststratification were pooled in groups of 5 for a total of 20 groups per replicate. These groups were tested for the presence of CMV via qRT-PCR as described above. The percentage of virus-infected seeds (*ST*) was estimated using a Poisson distribution as: *p* = 1 − (1 − *y/n*)*^1/k^*, where *p* is the probability of virus transmission by a single seed, *y* is the number of positive samples, *n* is the total number of samples assayed (*n* = 20), and *k* is the number of seedlings per sample (*k* = 5).

### 2.5. Data Treatment and Statistical Analyses

In the 165 Arabidopsis genotypes, we analyzed the average variance in *V* across plants of the same genotype. Within-genotype variance in this trait larger than two-fold the average variance in the whole set of genotypes was considered as indicative of an unreliable estimate of *V*. Nine Arabidopsis genotypes showed such inflated variance and were eliminated from the analysis and two more were deleted due to low number of replicates because of inoculation failure, and thus that the final dataset used for the GWAS contained 154 genotypes. We explored the phenotypic variation of *V* across Arabidopsis genotypes using the following general linear mixed model (GLMM): *V* = *μ* + Genotype + *ε*, where *μ* is the overall mean of the phenotypic data, “genotype” corresponds to the genetic differences among the selected Arabidopsis genotypes, and *ε* is the residual error term. Normality of the residuals was achieved through (*V + 2*)^5.36^ transformation (Kolmogorov–Smirnov test *p*-value = 0.143). The factor “genotype” was treated as a random factor.

Broad-sense heritability was estimated as *h*^2^_b_ = *V*_G_/(*V_G_* + *V_E_*), where *V*_G_ is the among-genotypes variance component and *V_E_* is the residual variance. Variance components were determined using GLMMs by the Restricted Maximum Likelihood (REML) method [44] as implemented in the R-library lme4 [45]. Statistical analyses were conducted using R version 3.6.0 [46].

### 2.6. Genome-Wide Association Study (GWAS)

The 154 Arabidopsis genotypes have been genotyped for 4,932,457 million single nucleotide polymorphisms (SNPs) evenly spaced across the genomes [35]. Following [39], only SNPs present in at least 55% of the genotypes and with minor allele relative frequency (MAF) > 0.03 were considered in this study, resulting in a total of 2,071,858 SNPs from which 88.6% were present in all accessions. GWAS was run for virulence using the FarmCPU (fixed and random model circulating probability unification) as implemented in the R package GAPIT v3 [47]. FarmCPU minimizes false positives by accounting for linkage disequilibrium between SNPs, which reduces model overfitting as compared to other methods [48]. To determine the optimal number of principal components to include, forward model selection using the Bayesian information criterion (BIC) was conducted. The Manhattan plot was constructed with R library rMVP [49]. Following previous studies [50,51,52], a threshold of −log_10_(*p*) ≥ 4 was considered to identify SNPs associated with natural variation of the trait measured in this study. Within this set of SNPs, we conservatively focused on those below the false discovery rate (FDR) threshold in order to minimize false positives/negatives. For our data set, FDR = 1 × 10^−8^. The functional annotation of SNPs was carried out using SnpEff v.4.1 [53] and the TAIR database v.10 [54]. Gene ontology (GO) annotation enrichment was tested with PANTHER v16.0 using the binomial test [55] and visualized using SimRel for semantic similarity measure with a threshold of *C* = 0.7, as implemented in REVIGO [56].

### 2.7. Bayesian Sparse Linear Mixed Model (BSLMM)

BSLMM as implemented in the GEMMA (genome-wide efficient mixed model association) software was used to infer the genetic architecture of CMV virulence in Arabidopsis [57], which allows testing whether the analyzed trait is determined by many loci of small effect (polygenic) or rather determined by a few loci of large effector (oligogenic). Monte Carlo Markov chains (MCMCs) were run for 10 million generations (recording every ten steps), with 10% discarded as burn-in. MAF cut-off was set at 3% and a normalized kinship matrix, more appropriate if SNPs with large effects have low MAF, was included. Normalized kinship matrix was estimated with Tassel 5.2.70 software [58]. The proportion of phenotypic variance explained by the available genotypes (PVE) was used as an estimator of the heritability of a given phenotypic trait. PVE is a flexible Bayesian equivalent of the narrow-sense heritability (*h*^2^). BSLMM also estimated the proportion of genetic variance explained by sparse effects (PGE); that is, the proportion of variance explained by loci with large effects.

### 2.8. Selection of SNPs Linking CMV Virulence and Seed Transmission Rate 

A Random Forest (RF) analysis was made to determine the SNPs associated with both CMV virulence and seed transmission rate. In this analysis, we used seed transmission rate estimates of 35 Arabidopsis genotypes with extreme virulence phenotypes as the response variable, and as the input, only the SNPs associated with CMV virulence as determined by GWAS (−log_10_(*p*) ≥ 4). Arabidopsis genetic group [39] and CMV virulence values were included as covariates (randomForest R package [59]). The model was run for 2000 trees and mtry = 2, as estimated with the trainControl function from the *caret* R package [60]. Significant association of each SNP with the response variable was estimated based on the empirical null distribution of SNPs with no importance in this variable following [61], as implemented in the *r2VIM* R package [62]. The relative importance of each SNP in CMV seed transmission rate was quantified as % increase in mean square error (MSE), that is, the increase in the error made by the RF in predicting the trait when the SNP is removed from the analysis.

## 3. Results

### 3.1. Natural Variation for CMV Virulence in Arabidopsis Genotypes 

**Figure 2:**
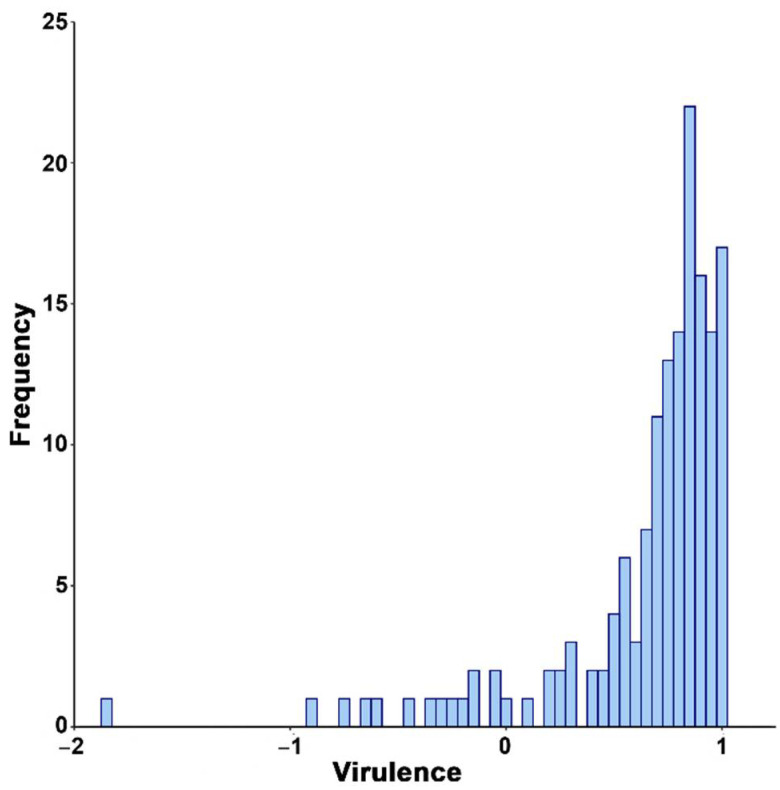
Frequency distributions of *Cucumber mosaic virus* (CMV) virulence in 154 *Arabidopsis thaliana* genotypes.

Overall, CMV-infected plants developed symptoms ranging from mild mosaic to leaf necrosis (Figure S1). Virus infection reduced plant progeny production, showing medium–high virulence in Arabidopsis genotypes (*V* = 0.637 ± 0.035; that is, infected plants produced on average 64% less seeds than mock-inoculated ones). However, virulence greatly varied across Arabidopsis genotypes, from plant sterilization (*V* = 1 in 13 genotypes) to overcompensation of the effect of infection on plant progeny production (negative values of *V* in 16 genotypes, with minimum values of −1.869 in genotype Lam-0) (Figure 2 and Table S1). This ample variation did not depend on the Arabidopsis genetic group (*F* = 1.801; *p* = 0.150) but did vary according to the plant genotype (*F* = 12.619; *p* = 1 × 10^−4^).

The broad-sense heritability of CMV virulence in Arabidopsis showed moderate–high values: *h*^2^_b_ = 0.68. Therefore, there was significant genetic variation among the studied Arabidopsis genotypes for the analyzed trait, such that the utilized population allowed a meaningful GWAS.

### 3.2. Genetic Architecture of Virulence in Arabidopsis

The 154 Arabidopsis genotypes accounted for 2.07 million SNPs, which were included in a BSLMM to evaluate how much of the variance in CMV virulence was explained by these SNPs. A moderate percentage of the phenotypic variance was explained by the genotyped SNPs [PVE: 51%; 95% ETPI (equal-tail probability intervals): 15–76%]. The strength of association of a SNP with CMV virulence above or equal to 0.25 was considered an indication of a large effect SNP that contributed the most to the phenotype. These large effect variants explained 58% (PGE: 58%; 95% ETPI: 32–96%) of the variance in CMV virulence. The number of SNPs with a large effect size was low (mean: 10; 95% ETPI: 4–18). Thus, the BSLMM indicated that Arabidopsis genetic architecture of CMV virulence involved few SNPs with detectable large effects.

**Figure 3:**
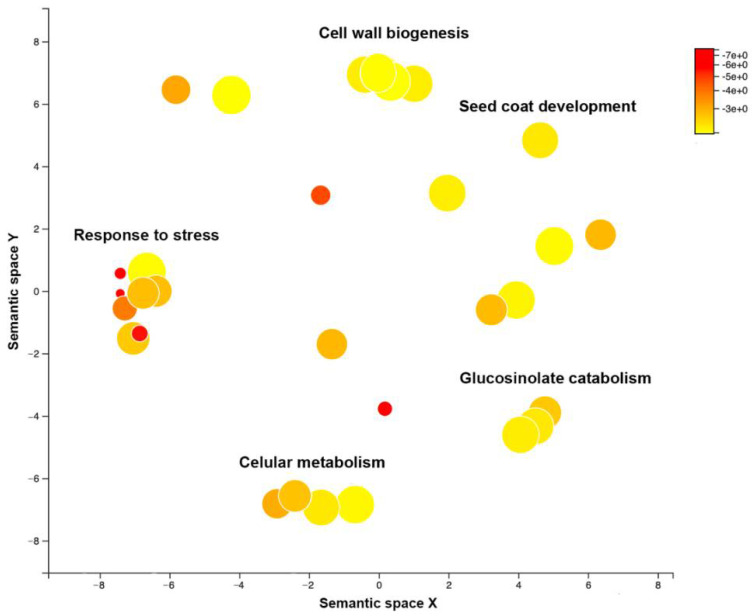
Scatterplot of enriched gene ontology (GO) terms for Arabidopsis determinants of CMV virulence. Color scale indicates significance of the term enrichment (−log_10_(*p*). Dot diameter indicates fold enrichment.

In parallel with the BSLMM, we performed a GWAS. The results indicated that 223 SNPs were significantly associated with CMV virulence in Arabidopsis (−log_10_(*p*) ≥ 4), with effect size varying from 0.08 to 0.42 (Table S2). To provide global insights into the biological processes associated with CMV virulence, we performed a GO term enrichment analysis based on the 223 detected SNPs (Figure 3). We identified 37 enriched GO terms (*p* < 1 × 10^−3^), which were reduced to 30 after eliminating redundant terms (Table S3). Similarity analyses of these 30 GO terms indicated that the largest clusters grouped functions related to response to stress (eight GO terms), cell wall biogenesis and cellular metabolisms (four GO terms each) and glucosinolate catabolism (three GO terms). GO terms in these four categories accounted for 63% of all enriched terms (Figure 3). Most other terms corresponded to general housekeeping functions (Table S3). One exception was SNP enrichment in genes related to seed coat development, a function highly related to progeny production (Figure 3).

**Figure 4:**
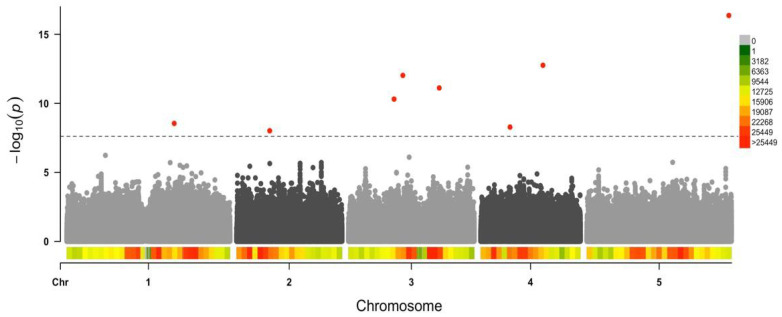
Manhattan plot illustrating the *Arabidopsis thaliana* genomic regions associated with CMV virulence. Dashed line indicates false discovery rate (FDR) threshold. Color scale represents single nucleotide polymorphism (SNP) density.

Only eight of the 223 SPNs passed the FDR threshold (FDR-adjusted *p*-value < 3 × 10^−3^ and −log_10_(*p*) ≥ 8) (Figure 4), representing most of the SNPs with the largest effects in virulence (effect size > 0.20) (Table S2). With few exceptions (nine SNPs, four of them affecting the same gene), the rest of the 215 SNPs had effects lower than 0.20. Therefore, only a reduced number of SNPs with large effects size were strongly associated with CMV virulence in Arabidopsis, in agreement with BSLMM.

Altogether, our analyses indicate that Arabidopsis genetic determinants of CMV virulence are mainly involved in functional categories associated with stress response and cell wall biogenesis. Of the identified SNPs, a few have detectable large effects and are strongly associated with the analyzed trait, whereas a larger number of SNPs are less strongly associated with virulence, most of them with smaller effect size.

### 3.3. Arabidopsis Genetic Determinants of CMV Virulence Identified by GWAS

We focused on the eight SNPs that passed the FDR threshold (Figure 4). MAF values for these SNPs varied between 0.08 and 0.11. For all of them, the minor variant had a negative effect on CMV virulence (Table 1). 

**Figure 5:**
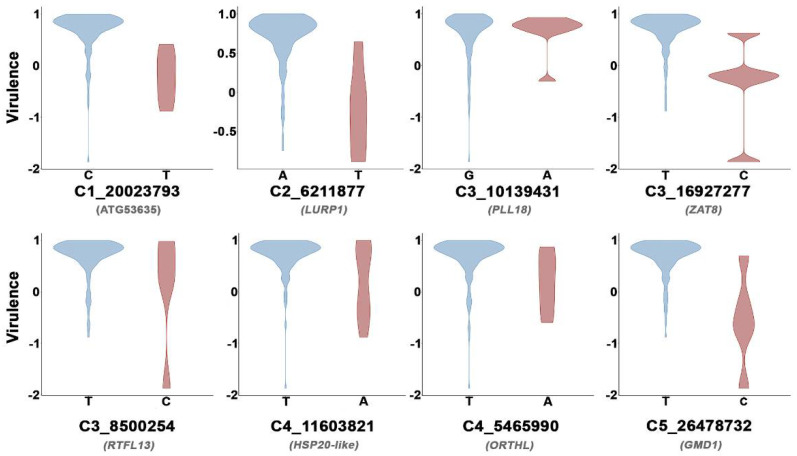
Distribution of CMV virulence across Arabidopsis genotypes harboring major (blue) and minor (red) variants of the eight SNPs strongly associated with this trait. Each SNP is indicated by the chromosome (C) and the position in which it is located, and the corresponding gene is shown in grey.

The two Arabidopsis SNPs with the strongest association with CMV virulence were located at genes encoding a GDP-d-mannose 4,6-dehydratase (GMD1) and a peptate lyase-like protein (PLL18) (FDR < 6.53 × 10^−7^). Both genes are involved in cell wall metabolism [63,64]. Of the remaining six SNPs strongly associated with virulence, three were involved in response to biotic and abiotic stresses: heat shock protein 20-like (HSP20-like), the zinc finger protein ZAT8 and the *late upregulated in response to Hyaloperonospora parasitica protein 1* (LURP1) [65,66,67,68,69]. Another two were related to different biological processes such as flowering time (ROTUNDIFOLIA like 13 protein, RTFL13) [70] and DNA methylation (ORTHRUS-like protein, ORTHL) [71], and one had unknown function (AT1G53635). To confirm the association of these SNPs with CMV virulence, we performed General Linear Models (GLMs) comparing CMV virulence in Arabidopsis genotypes with different variants in the eight detected SNPs. In all cases, analyses indicated significant differences (*F*_1,142–152_ ≥ 8.74; *p* < 3.6 × 10^−3^), supporting the role of these SNPs in the analyzed trait, with minor variants always showing a reduced virulence (Figure 5). 

In summary, the Arabidopsis SNPs strongly associated with CMV virulence are located in genes mostly involved in cell wall biogenesis and in stress response in agreement with GO term enrichment analyses, and minor variants of the SNPs in these genes generally reduce CMV virulence. 

### 3.4. Link between Arabidopsis Genetic Determinants of CMV Virulence and Seed Transmission

To analyze the potential genetic links between CMV virulence and seed transmission rate in the Arabidopsis genome, we selected 35 plant genotypes representing extreme virulence phenotypes in which we estimated the per cent of CMV seed transmission (*ST*) (Table S1). *ST* varied from absence of infected seeds (genotypes Cdc-3, Iso-4 and Lum-0) to 100% of infected seeds (genotypes Alm-0, Amu-0, Ang-0 and Aul-0). Then, we performed a RF analysis including the 223 SNPs associated with CMV virulence as predictors of virus seed transmission rate. The RF detected only seven of these SNPs as significantly associated with this trait (*p* < 0.041) (Table 2). None of the eight SNPs strongly associated with virulence was so with virus seed transmission rate.

**Figure 6:**
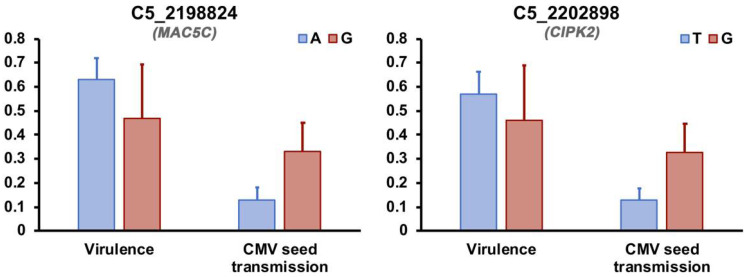
Distribution of CMV virulence and seed transmission rate across Arabidopsis genotypes harboring major (blue) and minor (red) variants of the two SNPs associated with both traits. Letters indicate the nucleotide at each SNP for the major and minor variants. Seed transmission rate is shown as *ST*/100 to fit in the same scale as virulence. Each SNP is indicated by the chromosome (C) and the position in which it is located, and the corresponding gene is shown in grey.

GLMs comparing CMV seed transmission rate in Arabidopsis genotypes with different variants in the seven SNPs indicated significant differences only for the two with the highest % MSE (*F*_1,34_ ≥ 8.74; *p* < 0.033) (Figure 6). The two SNPs associated with both CMV virulence and seed transmission rate were located in genes encoding the calcineurin B-like (CBL)-interacting protein kinase 2 (CIPK2) and the MOS4-ASSOCIATED COMPLEX SUBUNIT 5C protein (MAC5C). The former is a calcium regulated protein [72], and the latter has been reported as being involved in cell wall metabolism [73].

Hence, Arabidopsis genes involved in both CMV virulence and seed transmission rate control stress responses and cell wall metabolism.

## 4. Discussion

Most analyses of the host genetic determinants of plant virus virulence have identified genes involved in symptom development and/or plant susceptibility [8,11]. In contrast, genes associated with the effect of virus infection on plant progeny production are poorly characterized [74]. Identifying this genetic control is of great relevance to understand plant–virus interactions from an agronomic and ecological perspective: first, seed production is a key trait for wild plant population dynamics and a trait of agronomic importance [75,76]. Second, it modulates the chances for virus horizontal transmission (i.e., determines the number of available susceptible individuals) [1], and is central for viruses that disperse through seeds [27,77]. Here, through a GWAS including 154 plant genotypes, we identified Arabidopsis genes involved in the effect of CMV infection on seed production and we explored the potential link of these genes with the efficiency of virus seed transmission.

We identified SNPs in eight plant genes strongly associated with the effect of CMV infection on seed production. Three of these genes (*HSP20-like*, *ZAT8* and *LURP1*) were related to the response to biotic and abiotic stresses. The first two genes have been shown to be overexpressed in response to heat and salinity [78,79]. However, there is also evidence of their role in plant response to pathogens. For instance, many HSPs have been reported to alter their expression patterns upon infection by multiple pathogens [80]. Indeed, in *N. benthamiana* and rice HSP20 expression has been shown to change upon infection by *Impatiens necrotic spot virus* and *Rice stripe virus*, respectively, facilitating virus replication and within-host movement [68,81]. Similarly, Arabidopsis transcription factor *ZAT8* knock-out mutants have been reported to have higher susceptibility to *Pseudomonas syringae* [67], and in the same host other transcription factors of the ZAT family are responsive to infection by viruses, including CMV [82]. The third gene, *LURP1*, is also regulated in response to biotic stresses, being required for basal defense to *Hyaloperonospora arabidopsidis* [69]. Interestingly, our results are not the first evidence of a link between defenses to *H. arabidopsidis* and CMV. Another well-characterized resistance gene to this oomycete (*RPP8*) is allelic to *RCY-1*, which confers resistance to the isolate Y of CMV [83]. These results and ours suggest that *H. arabidopsidis* and CMV infection may trigger common defense pathways. In nature, Arabidopsis plants are commonly infected by both CMV and *H. arabidopsidis*, both pathogens imposing selective pressures on the host [21,23,84], such that common defense pathways would be selectively advantageous while minimizing energy investment at the same time. The evolutionary processes leading to this common solution is an interesting avenue of future research. It should be noted that the expression of *HSP20-like*, *ZAT8* and *LURP1* has been mostly studied in vegetative plant tissues, and their effect has been generally linked to reduced pathogen load and/or lower effect of infection on plant growth. The association of these three genes with the effect of infection on seed production might be explained if: (i) their differential expression in vegetative tissues affects plant reproduction, or (ii) these genes are also differentially regulated in plant reproductive organs. In any case, our results provide tentative evidence of a novel function for these genes in plant–virus interactions. 

Perhaps more interestingly, the SNPs with the strongest association, and the largest effect, with virulence were located in genes related to the cell wall metabolism (*GMD1* and *PLL18*). Current information on changes in the composition, structure and function of plant cell walls in response to infection has been largely restricted to non-viral pathogens [85,86]. The scant work on plant–virus interactions focused on the association between cell wall modifications and virus cell-to-cell and, to a lesser extent, systemic movement. For instance, pectin methylesterases and hydroxyproline-rich glycoproteins are needed for efficient *Tobacco mosaic virus* and *Potato virus Y* movement in tobacco and potato, respectively [87,88,89]. It could be argued that GMD1 and PLL18 work in a similar way: certain variants of these proteins might facilitate virus cell-to-cell and systemic colonization from entry points at plant leaves and therefore promote the invasion of reproductive structures. However, none of the two proteins encoded by these genes are significantly expressed in Arabidopsis leaves. Rather, their expression is largely restricted to flowers and pollen grains as they are mainly involved in the synthesis and remodeling of cell walls in these organs [63,64]. Hence, we hypothesize that CMV infection alters the functioning of these proteins, leading to the malformation of cell walls in flowers and/or pollen, which may affect plant fertility and therefore seed production. Arabidopsis genotypes with variants in these genes less prone to CMV effects would suffer less from infection. Further functional analyses of these GMD1 and PLL18 upon virus infection will allow testing this hypothesis. Regardless the mechanism involved, our results reveal a new role of the cell wall in plant–virus interactions as a regulator of the effect of infection on plant fitness. The identification of four large effect size SNPs that did not pass the FDR threshold but were significantly associated with CMV virulence within a gene encoding the prolyl 4-hydroxylase 11 (P4H11) (Table S2), which is also involved in cell wall biogenesis [90], and the results of our GO term enrichment, further support this conclusion.

The other two Arabidopsis genes strongly associated with CMV virulence were *RTFL13* and *ORTHL*. RTFL13 has been shown to be a regulator of flowering time that is expressed during cambium formation in Arabidopsis inflorescences [70]. There is ample evidence of the link between flowering time and the effect of virus infection on seed production [25,26,91]. Although the genetic bases are unknown, flowering genes are thought to control this process [18]. In agreement, *RTFL13* would be a candidate gene to be involved in such control. As for ORTHL, an epigenetic regulator of embryogenesis [92], any effect of virus infection on embryo development would directly impact plant fitness. Our results suggest that such effects would occur through alterations of the epigenetic regulation of plant reproduction. Indeed, virus infections have been repeatedly shown to alter plant epigenetics (including DNA methylation) [93,94]. Hence, the identification of these two genes as potential regulators of CMV virulence support the idea that at least part of the host control of this trait occurs at the plant reproductive stage, and independently of the virus effects on vegetative structures.

Although none of the Arabidopsis SNPs strongly associated with CMV virulence were also modulating virus seed transmission rate, our RF analysis identified two SNPs that were linked to both traits: CIPK2 and MAC5C. CIPK2 is a protein kinase associated to calcineurin B-like protein (CBLs), which is a group of calcium sensors. In plants, calcium fluxes are important for sensing a variety of stimuli, including abiotic conditions and pathogens, and in these processes both CBLs and CIPKs are involved [72,95]. For instance, tobacco CIPK2 has been shown to be involved in tolerance to drought [72]. Although in Arabidopsis the role of CIPK2 is not known, other CIPKs (such as CIPK6) have been demonstrated to induce plant resistance to pathogens when silenced. Thus, it could be hypothesized that CIPK2 acts in a similar way in response to CMV. Interestingly, CIPK2 is highly expressed in flowers [96], which may explain its common role in virulence and seed transmission: the differential expression of CIPK2 may induce local resistance to the virus in flowers, minimizing the effects of infection in seed production and preventing seed invasion. Similarly, MAC5C, part of the secondary cell wall regulation network [73], is expressed during seed germination [97]. In infected seeds, MAC5C may contribute to minimize the effect of CMV presence on secondary wall metabolism, promoting seed viability and favoring virus seed transmission. As part of the MAC complex, MAC5C also has a role in plant resistance to pathogens [98]. The specific mechanism by which MAC5C is involved in plant defenses is not well characterized [99]. However, this work was restricted to analysis in plant vegetative structures. Similarly to CIPK2, MAC5C could also influence virulence and seed transmission rate by restricting virus invasion of reproductive organs. It is worth mentioning that here we did not quantify CMV load in the reproductive plant organs, which would have contributed to understand whether certain variants of these two genes prevent virus invasion. On the other hand, we show that variants with lower virulence had higher seed transmission rate, suggesting a negative correlation between both traits, which is in agreement with our previous results [28], strengthening the evidence of a role of these genes as multi-trait regulators.

In summary, this work identifies genes related to stress response and cell wall metabolism as potential regulators of the effect of virus infection on plant progeny production and of virus seed transmission rate. Although functional analyses are needed to validate these results, our GWAS provides tentative evidence of novel roles for these plant genes in plant–virus interactions, opening a new avenue of future research.

## Figures and Tables

**Table 1:** SNPs significantly associated with virulence according to FDR threshold.

SNP ^a^	Protein	Description	FDR	Effect
C5_26478732	GMD1	Cell wall metabolism	8.92 × 10^−11^	−0.42
C3_10139431	PLL18	Cell wall metabolism	6.53 × 10^−7^	−0.40
C4_11603821	HSP20-like	Abiotic stress response	1.81 × 10^−7^	−0.22
C3_16927277	ZAT8	Biotic and abiotic stress response	3.96 × 10^−6^	−0.30
C3_8500254	RTFL13	Flowering time	2.05 × 10^−5^	−0.23
C1_20023793	AT1G53635	Unknown	9.81 × 10^−4^	−0.23
C4_5465990	ORTHL	DNA methylation	1.56 × 10^−3^	−0.21
C2_6211877	LURP1	Biotic stress response	2.51 × 10^−3^	−0.22

^a^ Each SNP is named by the chromosome (C) in which it is located and its position in the chromosome.

**Table 2:** SNPs significantly associated with CMV seed transmission rate according to Random Forest (RF). CIPK2: calcineurin B-like (CBL)-interacting protein kinase 2; JAL4: Jacalin-related lectin 4; MAC5C: MOS4-ASSOCIATED COMPLEX SUBUNIT 5C protein. MEG: Maternally expressed gene.

SNP ^a^	Protein	Description	*p*-Value	% Inc. MSE ^b^
C5_2202898	CIPK2	Response to abiotic stress	0.012	8.11
C5_2198824	MAC5C	Cell wall metabolism	0.012	2.77
C5_11777493	AT5G31963	Unknown	2 × 10^−16^	0.83
C2_7038732	MEG	Embryogenesis	2 × 10^−16^	0.73
C3_15811438	AT3G44030	Unknown	0.041	0.72
C1_12253373	JAL4	Response to biotic stress	0.041	0.53
C1_16528990	AT1G43750	Unknown	0.033	0.49

^a^ Each SNP is named by the chromosome (C) in which it is located and its position in the chromosome. ^b^ Per cent of increase in the mean square error (MSE).

## Data Availability

Data available as supplementary material.

## Supplementary Materials

The following are available online at https://www.mdpi.com/article/10.3390/microorganisms9040692/s1, Figure S1: Symptoms induced by CMV infection in Arabidopsis. Upper line shows mock-inoculated plants and lower line CMV-infected plants of Arabidopsis genotypes Cem-0 (A and E), Gra-0 (B and F), Ses-0 (C and G) and Vas-0 (D and H). Table S1: List of Arabidopsis genotypes utilized in the GWAS and average virulence (*V*) and seed transmission rate (*ST*) values per genotype; Table S2: SNPs in the Arabidopsis genome associated with CMV virulence; Table S3: GO enriched terms in the 223 host SNPs associated with CMV virulence in *Arabidopsis thaliana*.
