# Supplementary material for: Arabidopsis thaliana Genes Associated with Cucumber mosaic virus Virulence and Their Link to Virus Seed Transmission
Source: Microorganisms. 2021 Mar 27;9(4):692. doi: 10.3390/microorganisms9040692 (PMC8067046; doi:10.3390/microorganisms9040692)
Supplement: Supplementary file 1 [file microorganisms-09-00692-s001.zip › Figure S1.docx]

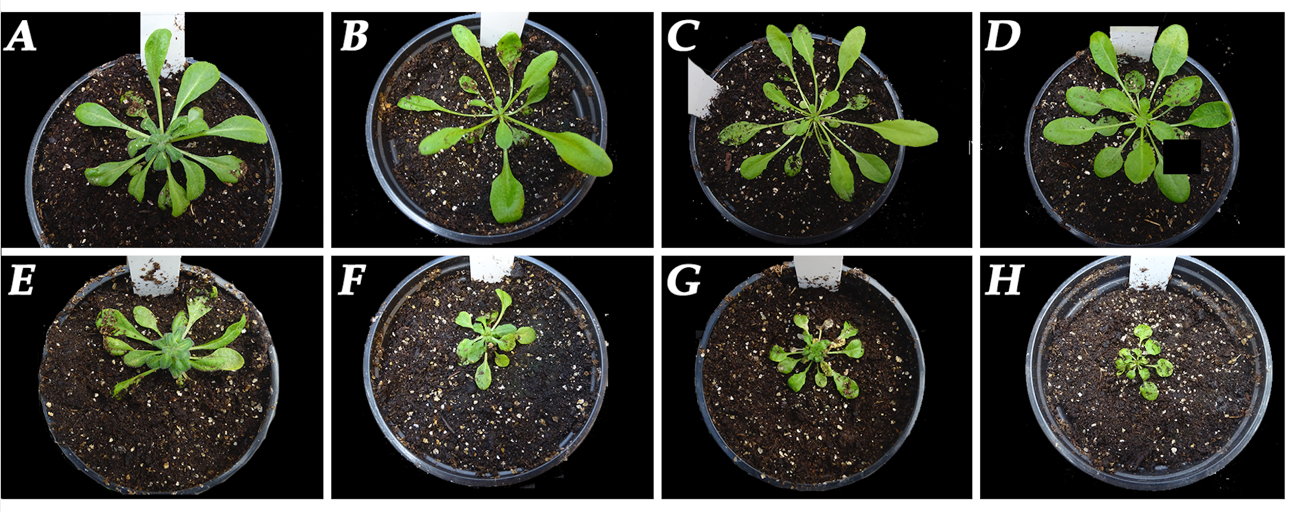


**Figure S1.** Symptoms induced by CMV infection in Arabidopsis. Upper line shows mock-inoculated plants and lower line CMV-infected plants of Arabidopsis genotypes Cem-0 (A and E), Gra-0 (B and F), Ses-0 (C and G) and Vas-0 (D and H).
